# Detection of the Soluble Solid Contents from Fresh Jujubes during Different Maturation Periods Using NIR Hyperspectral Imaging and an Artificial Bee Colony

**DOI:** 10.1155/2019/5032950

**Published:** 2019-04-01

**Authors:** Haixia Sun, Shujuan Zhang, Caihong Chen, Chengji Li, Shuhai Xing, Jianglong Liu, Jianxin Xue

**Affiliations:** College of Engineering, Shanxi Agricultural University, Taigu 030801, China

## Abstract

To perform accurate and synchronous detection of the soluble solid contents (SSC) in fresh jujubes at different stages of maturity, hyperspectral imaging was used to establish robust models. The combined data constituting four maturation stages were used to build the grid-search least squares support vector machine (GS-LS-SVM) model. The determination coefficient (Rp^2^), the root-mean-square error (RMSEP), and the residual predictive deviation (RPD) of the prediction set for samples of the overall stages were 0.98, 1.10%, and 7.85, respectively. Furthermore, a successive projections algorithm (SPA) was used to extract the characteristic wavelengths of the combined data. An artificial bee colony (ABC) algorithm (for the prediction set, Rp^2^ = 0.98, RMSEP = 1.19%, RPD = 7.25) was used to improve the SPA-LS-SVM model, which was better than the SPA-GS-LS-SVM model (for the prediction set, Rp^2^ = 0.98, RMSEP = 1.24%, RPD = 6.96). Lastly, visualization of the SSC distribution map was performed based on the SPA-ABC-LS-SVM model, which clearly showed that the SSC gradually increased during maturation. The results indicated that it was realistic to construct a detection model of the multimaturity stage. This research also demonstrated that the combination of hyperspectral imaging and the ABC had good application values in the testing of agricultural products.

## 1. Introduction

Jujubes are rich in many compounds, such as sugar, vitamins, amino acids, and minerals. Jujubes have high nutritional and medicinal value. The soluble solid content (SSC) is a key parameter in the quality assessment of this fruit, and it influences taste and is directly related to the purchasing willingness of consumers. The SSC is also an important indicator of the physiological changes that occur in fruits during ripening and for determining the harvest time [1, 2]. Therefore, the detection of the SSC in fresh jujubes during maturation has an important value in terms of increasing the added value and meeting the ever-increasing consumer demand for this fruit. Near-infrared (NIR) spectroscopy (12500–4000 cm^−1^) has the advantages of fast, nondestructive, and no complicated preprocessing of samples. NIR spectroscopy reflects the characteristics of molecular frequency doubling and combined frequency absorption. The mid-infrared (MIR) spectroscopy (4000–400 cm^−1^) is the main molecular vibration absorption region, which contains large amount of information and good fingerprint characteristics. MIR spectroscopy has better resolution to single species than NIR spectroscopy. NIR also has shortcomings such as wide frequency band and serious information overlap. However, compared with MIR, NIR can be easily and quickly completed by diffuse reflection fiber in spectral acquisition, and has lower instrument cost. It was widely used in the nondestructive testing of food fat, sugar, and other qualities [3, 4]. Conventional NIR spectrometers only acquire the spectral information at a specific point and use it as representative information for the sample. Because of the advantage of image-spectrum merging, hyperspectral imaging (HSI) technology can be used to obtain the information from any point in a 3D space, which is impossible to achieve with a traditional spectrometer. However, the instrument cost of HSI technology is higher. Compared with the NIR spectrometer, the HSI is more accurate for positioning in the measurement of the sample and can obtain more abundant information. The content of components at each pixel can be predicted by a multivariate model based on HSI and converted to a physicochemical image. The obtained image shows the spatial distribution of the chemical components in the test sample. HSI technology had been widely applied to the quality detection in fruits [5, 6]. During jujube maturation, the sugar content clearly changed and was the primary factor used for assessing the maturity and the change in quality [7–9]. Therefore, NIR hyperspectral imaging techniques were used to establish accurate calibration models and visualize the spatial distribution of SSC within the sample and the changes in different maturity stages.

During the quality detection of jujube fruits by spectrum, the external qualities (such as bruises, insect infestations, and crack features) and internal qualities (such as SSC and hardness) were researched [10–14], and the resulting models were primarily established by individual maturity stage. However, the fruit ripening process was influenced by differences in external factors, such as light, nutrients, temperature, flowering time, and environmental conditions in different fruit-bearing branches [15–17]. The harvested fruit is the product of all the maturity stages. In addition, one of the important issues in this measurement is model robustness during spectral detection. The predictive ability of the established calibration model was affected by different instruments and different production systems [18, 19]. During the identification of external defects (bruises, insect infestations, and cracks), the detection abilities of 2 instruments were compared by Wu et al. [20]. Mireei and Sadeghi [21] reported that a better result for classifying bunch withering disorder was obtained for the late harvest dates than those from the combined data and normal harvested dates. However, synchronous detection of SSC at different maturity stages was rarely studied. Moreover, improving the robustness of a prediction model and investigating the distribution characteristics of the SSC in samples from different maturity stages were not extensively researched.

The least squares support vector machine (LS-SVM) has been successfully applied to the spectral detection of fruit quality. However, the generalization ability of the LS-SVM model is largely determined by the regularization parameter “*γ*” and the bandwidth of the Gaussian radial basis kernel “*σ*.” At present, metaheuristic algorithms have been applied to the optimization of single/multiobjectives, such as genetic algorithms [22], particle swarm optimization algorithms [23], and ant colony algorithms [24]. Compared with the above methods, the artificial bee colony (ABC) only used the fitness function as the basis of evolution, and it involved a simple operation, low control parameters, strong robustness, and the ability to easily get rid of the local optimal solution and attained a relatively better solution quality [25–27]. An ABC was used for solving the optimal solution in numerical problems. During the optimization of the NIR detection model for fruit, different preprocessing methods, different selection algorithms with sensitive bands, and different stoichiometry algorithms were primarily used as the starting point. However, the predicted results were seldom analyzed by optimizing the parameters of the modeling method.

Therefore, the objective of this research was to improve the predicted accuracy of SSC detection in fresh jujubes at different maturity stages and to clarify the distribution and variation in the SSC by combining spectral and spatial information. The specific objectives of the study were as follows: (1) to evaluate changes in the SSC and spectral characteristics at different maturity stages of the “Huping” jujube; (2) to investigate the SSC prediction ability for samples from different maturity stages; (3) to use SPA for data reduction, optimize LS-SVM using ABC, compare and analyze the predicted accuracy combined with the grid-search LS-SVM, and develop an accurate detection model of SSC; (4) to establish the visual distribution map of SSC at different maturity levels and explain the distribution and variation in the SSC.

## 2. Materials and Methods

### 2.1. Sample Preparation

**Figure 1:**
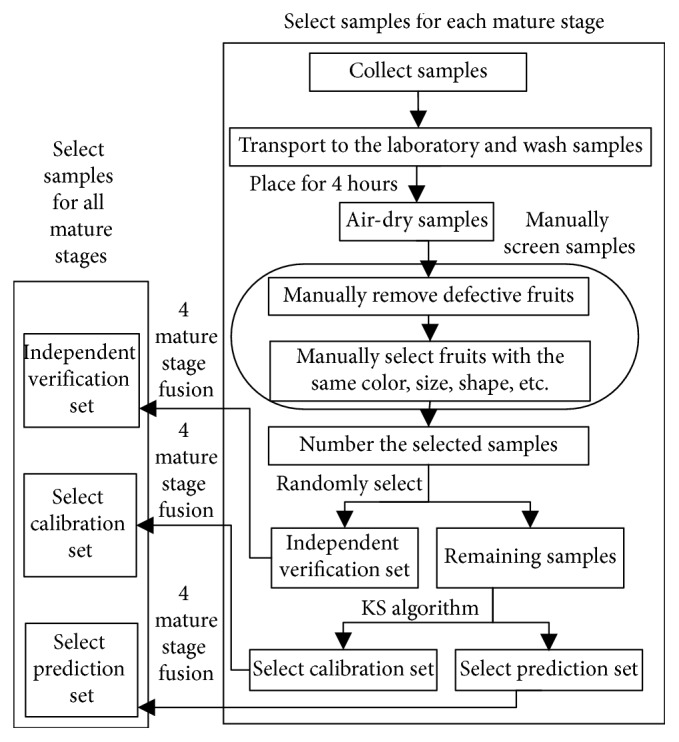
Workflow showing the selection of the samples.

160 fresh jujubes (cv. “Huping zao”) were collected, respectively, at immature, white-mature, crisp-mature, and full-mature stages in 2017 from Xiaobai orchard, which is located in Taigu, China. All the samples were packed in airtight polyethylene bags and transported to the laboratory on the same day as the sampling. The workflow of the sample selection is shown in Figure 1. To avoid the effects of surface contamination on the fruit, the fresh jujubes were washed. Furthermore, all the samples were placed under laboratory conditions for 4 hours to air-dry them and restore them to an indoor temperature and humidity environment. The samples were then screened. The color, size, and shape of the fruits were consistent to reduce the impact of individual differences on the testing results between samples from the same maturity stage, and heterocarpous and damaged fruits were removed. The final sample size for each maturity became 150 samples. Finally, the selected samples were numbered. Thirty samples in each mature stage were randomly selected and used as independent verification sets. The remaining samples were divided into calibration sets (90 samples) and prediction sets (30 samples) for each mature stage by Kennard–Stone (KS) algorithm [28]. Therefore, 600 samples were used for the experiment in this study, with 360 samples for the calibration set, 120 samples for the prediction set, and 120 samples for the independent verification set.

### 2.2. Hyperspectral Image Acquisition and Calibration

In the experiment, the “Gaia Sorter” hyperspectral sorting instrument (Zolix Instruments Co., Ltd., Beijing, China) with a wavelength range of 900–1700 nm was used to collect the NIR spectra and images. The instrument is composed of an Image-*λ*-N17E spectral camera with an InGaAs detector, an illumination unit with four 35 W bromine tungsten halogen lamps, a dark chamber, a mobile platform, and a computer (Lenovo (Beijing) Co., Ltd., Beijing, China) with SpecView software. To avoid image distortion and information oversaturation, the time of exposure was 0.13 s, the distance between the sample and the lens was 220 mm, and the moving speed of the mobile platform was 7.0 mm/s. The black and white correction was performed using the following equation before collecting the spectrum [20], where *R* is the corrected image, *I*_r_ is the original image, *I*_d_ is the image of the blackboard correction, and *I*_w_ is the image of the whiteboard correction:

(1) $$\begin{array}{c} R=\frac{I_{\text{r}}-I_{\text{d}}}{I_{\text{w}}-I_{\text{d}}}. \end{array}$$

### 2.3. Measurement of Soluble Solid Contents

The real SSC of each jujube was determined by handheld refractometer (Chengdu Haochuang Photoelectric Instrument Co., Ltd., Chengdu, China) with temperature compensation, and the results were expressed in %. The SSC at the equatorial position of each sample was determined.

### 2.4. Chemometric Methods

#### 2.4.1. Successive Projections Algorithm

The successive projections algorithm (SPA) is a simple and efficient algorithm for characteristic wavelength extraction, and it not only eliminates the problem of multicollinearity between characteristic wavelengths but can also prevent the repeated extraction of overlapping variants [10, 29].

#### 2.4.2. Artificial Bee Colony and Least Squares Support Vector Machines

The LS-SVM [10, 30] is an improved algorithm of support vector machines, performs linear decomposition, and constructs the optimal linear function by mapping the nonlinear vectors in the original space to high dimensional space, reducing the computational complexity. Based on the intelligent foraging behavior of honeybees, a swarm intelligence algorithm named ABC algorithm was proposed and used for the intelligent search and optimization of parameters by Karaboga [31]. In the paper, the RBF was used as the kernel function. The traditional grid-search (GS) based on leave-one-out cross-validation and an ABC were used to find the best parameters, respectively. According to the relevant research [32, 33] and previous studies, the search range of *γ* and *σ*^2^ was set to [1, 5000]. The optimizing and modeling steps using the ABC were as follows:

1. A data reduction was performed by SPA, and then the selected variables were input into the ABC-LS-SVM.
2. Initialize parameter: the swarm size was 60. The number of food sources (SN) was 30, which was equal to the number of employed bees. The maximum number of searches for honey (limit) was 120. The maximum number of iterations was 30. The dimension of the solution vector (*γ* and *σ*^2^) was 2.
3. Food sources (*x*_*ij*_) were initialized randomly, where *x*_*ij*_ is the *j*th element of *x*_*i*_ and *x*_*i*_ is the position of the *i*th food source. The fitness of the *i*th food source (fit_*i*_) was calculated using the following equation:

(2) $$\begin{array}{c} {\text{fit}}_{i}=\left\{\begin{array}{cc} \frac{1}{\left(1+f_{\text{obj}}\right)}, & f_{\text{obj}}\geq \text{0,}\\ 1+\text{abs}\left(f_{\text{obj}}\right), & \text{otherwise}. \end{array}\right.\; \end{array}$$

The optimized objective function (*f*_obj_) was the mean squared error (MSE) of the prediction set. The smaller the MSE was, the better the solution was. The MSE was calculated using the following equation:

  (3) $$\begin{array}{c} \text{MSE}=\sum \frac{{\left(y_{\text{t}}-y\right)}^{2}}{n}, \end{array}$$
  where *n* is the number of fruit samples used in the calculation, *y* is the measured value of the SSC, and *y*_t_ is the calculated value of the SSC.
(4) In the vicinity of the *x*_*i*_, employed bees searched and created a new food source. The new solution would be saved if the fitness value of the new solution was better than that of the original solution. Instead, the new solution was abandoned. The probability of each solution (*P*_*i*_) was calculated using the following equation:
  (4) $$\begin{array}{c} P_{i}=\frac{0.9\;*\;{\text{fit}}_{i}}{\max\left({\text{Fit}}_{i}\right)}+0.1, \end{array}$$
  where max (Fit_*i*_) is the maximum value in *i* fitness values. Each onlooker bee chose the food source according to the probability, calculated the fitness value, and saved the current optimal solution.
(5) If the current food source was accessed more than the upper limit, then the current food source would be abandoned, employed bees would turn into scout bees, and scout bees would randomize their own location and start to search. Otherwise, the optimal solution that was generated was output by this ABC algorithm.
(6) The ABC algorithm was run 10 times, the results of multiple runs were saved, and the robustness of the program was analyzed. The optimal solutions of *γ* and *σ*^2^ were determined, and then the ABC-LS-SVM prediction model was established.

#### 2.4.3. Evaluation of Model Performance

The calibration and predictive ability of the model were assessed using the determination coefficient of the calibration set (Rc^2^), the determination coefficient of prediction (Rp^2^), the root-mean-square error of calibration set (RMSEC), the root-mean-square error of prediction (RMSEP), and the residual predictive deviation (RPD), bias, and slope.

#### 2.4.4. Chemical Imaging Processing

To observe the changes in the soluble solids in the sample, visual maps of the SSC distribution were needed. The specific steps for establishing a visual distribution map were as follows: First, the spectral and spatial information from each pixel were extracted in hyperspectral images of fresh jujubes. Then, based on the best calibration model, the SSC of each pixel could be predicted using the obtained spectral information. Last, combining the spatial information of each pixel in the sample, a 2-dimensional image was rebuilt using the predicted value of the SSC. In this study, the color parameter 0–60 indicated that the SSC was 0%–60%.

## 3. Results and Discussion

### 3.1. Analysis of Quality Parameters

The statistical values of the SSC in the jujubes are shown in Table 1. At the immature and white-mature stages, the SSC was lower. The SSC significantly increased in the crisp-mature stage, and the growth rate (37.53%) was the fastest. The mean value of the SSC reached 35.94% at the full-mature stage. During the growth of the fresh jujubes, the SSC gradually increased, and the difference in the SSC was significant at different maturity stages, especially as the fruits ripened. Similar results were found by Park and Kim [8] and Moradinezhad et al. [7]. This result was primarily caused by the accumulation of monosaccharides and disaccharides (increased glucose and fructose contents) during the maturation process [8, 9]. On the whole, the calibration set has a wider range of SSC distributions than the predicted set.

### 3.2. Analysis of Spectra

**Figure 2:**
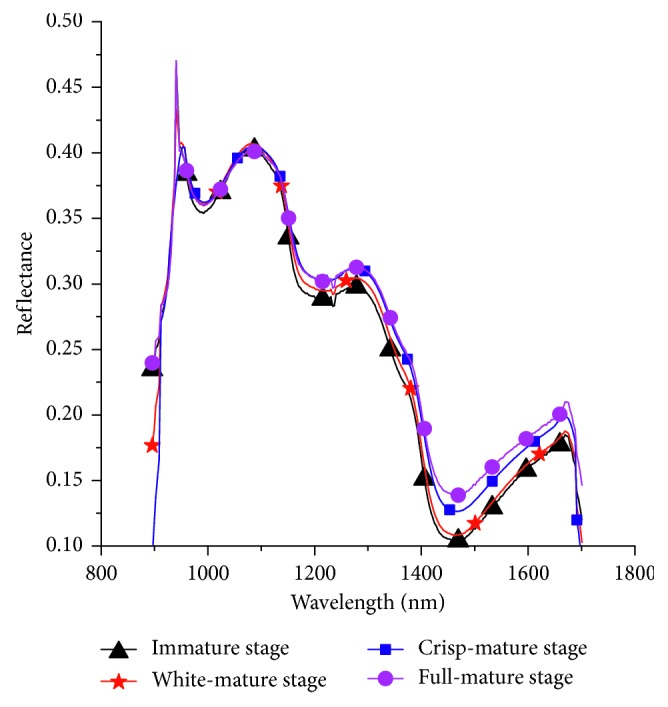
Average spectral curves of the samples.

The spectral information of the 15 ∗ 30 pixel area at the equatorial position of the sample was extracted using ENVI (ITT Visual Information Solutions, Boulder, CO, USA). The mean value was calculated and used as the spectral information for each sample. The average spectral curve is presented in Figure 2. The spectra in the 950–1675 nm range were selected for the following analysis, owing to the large amount of noise in the 900–950 nm and 1675–1700 nm ranges. The spectra of the four mature stages were consistent. An obvious absorption peak at 980 nm was caused by stretching the O-H vibration in the water molecules. There was an obvious combination frequency absorption peak of water molecules at approximately 1224 nm. There was an obvious absorption peak near 1450 nm, which was related to the presence of O-H stretching first overtone [34, 35]. Travers et al. [36] reported that 1450 nm was related to the prediction of SSC rather than dry matter, and there was a C-H stretching vibration near 1600 nm and 1700 nm. In the 1377–1672 nm range, the reflectivity of the fruit was obviously different during the four maturation stages, and a gradual declining trend was presented, which might be related to the changes in the SSC.

### 3.3. Predicting the SSC at Different Ripening Stages Using the Full Spectral Range

To study the prediction performance of the model for samples from different maturity stages, samples from the calibration set at the four maturation stages were combined, and the combined full-band spectral information was used as input of the GS-LS-SVM model. The optimal values of the parameters (*γ* and *σ*^2^) and the prediction results are shown in Table 2. Good predicted results were obtained for samples from 4 single maturity stages (Rp^2^ = 0.81–0.85, RMSEP = 0.64%–1.50%, RPD = 2.03∼2.31). The Rp^2^, RMSEP, and RPD for samples of the overall stages were 0.98, 1.10%, and 7.85, respectively. Therefore, the performance of the calibration model was robust and accurate, and it was practical to build the detection model in the multimaturity stage.

### 3.4. Predicting the SSC Using Important Wavelengths and ABC-LS-SVM

Based on the combined data from the four maturity stages in the range of 950–1675 nm, the SPA was used for data reduction because of the information redundancy over the full spectral range. Twenty-four characteristic wavelengths (1584, 1599, 1170, 1352, 1243, 1501, 1157, 1141, 1075, 1511, 1441, 1011, 1618, 1390, 1406, 1542, 1482, 1431, 1644, 1612, 1577, 976, 957, and 953 nm; those wavelengths are listed in descending order of importance) were selected by the SPA when the root-mean-square error was 2.018%. 1169.4–1314.5 nm (3 v) and 1199.3–1348.2 nm (3 v) were associated with the C-H stretch vibration, 1620–1800 nm was related to the 1st frequency of the C-H stretch vibration, 1397.4–1507.0 nm (2 v) and 1436.7–1571.4 nm (2 v) were related to the O-H stretch vibration, there was a stretching vibration of C=O near 1507.2–1739.1 nm (4 v), and 967.2–1095.2 nm (3 v) was related to the O-H stretch vibration [37–39]. Therefore, 24 characteristic wavelengths were correlated with the C-H, C=O, and O-H stretch vibration.

**Figure 3:**
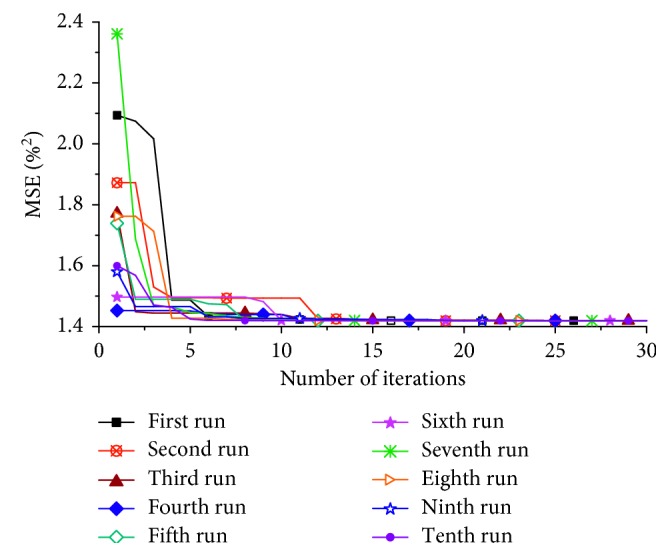
Changing trend of the MSE in the ABC-LS-SVM model searching for optimal parameters.

Based on the characteristic wavelengths selected by SPA, an ABC algorithm was used to search for optimal parameters in the hyperparameter space of LS-SVM model. The curve of MSE in the iterative process is shown in Figure 3. In 10 ABC runs, the minimum values of MSE were all around 1.42%^2^, the obtained optimal parameter values and the number of iterations at the time of convergence were respectively similar, which indicated that the method had good robustness. In the third run, the MSE converged to 1.42%^2^ at the 11th iteration; the optimal values of *γ* and *σ*^2^ were, respectively, 5.00 × 10^4^ and 49.52.

The GS-LS-SVM and ABC-LS-SVM were used to build models, respectively. The results are displayed in Table 3. The Rp^2^, RMSEP, and RPD for the prediction set using GS-LS-SVM model were 0.98, 1.24%, and 6.96, respectively. According to the results of prediction set, modeling accuracy and stability have been effectively improved by the ABC-LS-SVM method (Rp^2^ = 0.98, RMSEP = 1.19%, RPD = 7.25). Then, an independent verification set was used for the verification of models. Compared with the GS-LS-SVM model (Rp^2^ = 0.97, RMSEP = 1.48%, RPD = 6.09), the ABC-LS-SVM model got better predictions results (Rp^2^ = 0.98, RMSEP = 1.37%, RPD = 6.58). ABC's prediction effect on prediction set and verification set was significantly better than grid-search method, and ABC-LS-SVM had better fitting accuracy and generalization ability than GS-LS-SVM. Consequently, ABC can be used to improve the predicted performance based on LS-SVM, and it has a certain engineering application value.

**Figure 4:**
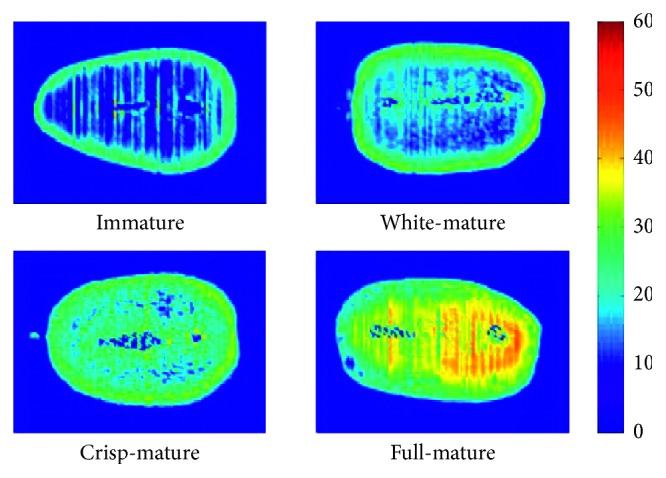
Examples of visualization in an SSC distribution map of the 4 maturity stages.

During the maturation process, the internal components and contents changed, and the SSC in each pixel of the samples was difficult to determine by conventional chemical methods. Based on 24 characteristic wavelengths selected by SPA, the spectral information corresponding to 24 characteristic wavelengths in each pixel of the samples was extracted and used as the input of the ABC-LS-SVM model, and the SSC of each pixel was predicted. The visual distribution of the SSC in the four maturity stages is established in Figure 4, with a colored bar displaying different colors from blue (low value) to red (high value). The color of the immature fresh jujube was primarily blue, and individual pixels are shown in yellow, which indicated that the SSC was low. The color of the full-mature fresh jujube was primarily yellow-red, the individual pixels were blue and cyan, the SSC was high, and the maximum value was 50.23%. With the advancement of the maturation process, the density of the yellow color gradually increased, which indicated that the SSC increased. The distribution of SSC at different locations in each sample was asymmetric and nonuniform. These phenomena were primarily attributed to the different variation speeds for chemical compounds in fresh jujubes. Therefore, the visual map of the distribution was useful for analyzing the distribution and transformation of the SSC during the maturation process of fresh jujubes.

## 4. Conclusions

To evaluate the internal quality accurately and robustly, the detection models of the SSC in fresh jujubes at different maturity stages (immature, white-mature, crisp-mature, and full-mature stage) were established using hyperspectral imaging technology. The GS-LS-SVM model made with combined data constituting the four maturity stages obtained accurate prediction results (Rp^2^ = 0.98, RMSEP = 1.10%, RPD = 7.85). It was realistic to construct the detection model using multimaturity stages. Twenty-four characteristic wavelengths of the combined data from the four maturity stages were extracted by SPA, and the SPA-ABC-LS-SVM model yielded more accurate results (Rp^2^, RMSEP, and RPD for the prediction set were 0.98, 1.19%, and 7.25, respectively; Rp^2^, RMSEP, and RPD for the independent verification set were 0.98, 1.37%, and 6.58, respectively) than the SPA-GS-LS-SVM model (Rp^2^, RMSEP, and RPD for the prediction set were 0.98, 1.24%, and 6.96, respectively; Rp^2^, RMSEP, and RPD for the independent verification set were 0.97, 1.48%, and 6.09, respectively). The method for optimizing the LS-SVM parameters using ABC could improve the predicted accuracy in the SSC assessment. The SSC of each pixel in the samples was predicted, and the visualization of the SSC distribution map was accomplished based on the SPA-ABC-LS-SVM model. During fruit maturation, the SSC gradually increased, but the growth rate was different and the distribution of SSC at different locations in each sample was asymmetric and nonuniform. This study provides a theoretical basis and method for the grading and quality sorting of fresh jujubes.

## Figures and Tables

**Table 1:** Statistics for the SSC (%) at different ripening stages of “Huping” jujubes.

Ripeness stage	Data set	Max. (%)	Min. (%)	Mean (%)	Standard deviations (%)
Immature stage	Total samples	17.8	9.1	13.61	2.55
	Calibration set	17.8	9.1	13.89	2.53
	Prediction set	17.2	10.4	14.05	1.98
	Verification set	17.4	9.2	12.35	2.78

White-mature stage	Total samples	22.9	16.0	19.85	1.35
	Calibration set	22.9	16.0	19.88	1.26
	Prediction set	22.4	16.7	19.71	1.30
	Verification set	22.4	16.1	19.93	1.66

Crisp-mature stage	Total samples	37.6	18.8	27.30	3.96
	Calibration set	37.6	18.8	27.49	4.30
	Prediction set	36.2	20.2	26.96	3.46
	Verification set	35.8	21.7	27.10	3.41

Full-mature stage	Total samples	44.0	24.2	35.94	3.33
	Calibration set	44.0	28.3	36.20	3.14
	Prediction set	41.9	31.0	36.04	2.74
	Verification set	41.2	24.2	35.05	4.25

Overall stage	Total samples	44.0	9.1	24.18	8.86
	Calibration set	44.0	9.1	24.37	8.90
	Prediction set	41.9	10.4	24.19	8.63
	Verification set	41.2	9.2	23.61	9.01

**Table 2:** Results of the GS-LS-SVM models for the prediction of SSC in different maturation stages of jujube samples over the full spectral range.

Prediction set	*γ*	*σ* ^2^	Rc^2^	RMSEC (%)	Rp^2^	RMSEP (%)	Slope	Bias	RPD
Immature	1.21 ∗ 10^4^	1.21 ∗ 10^3^	0.95	1.35	0.85	0.86	1.03	0.14	2.30
White-mature					0.83	0.64	1.08	0.06	2.03
Crisp-mature					0.84	1.50	0.79	0.60	2.31
Full-mature					0.81	1.21	0.82	−0.33	2.26
Overall					0.98	1.10	0.97	−0.08	7.85

**Table 3:** Results of GS-LS-SVM and ABC-LS-SVM models for the prediction of SSC using important wavelengths.

Modeling algorithms	Data set	*γ*	*σ* ^2^	Rc^2^	RMSEC (%)	Rp^2^	RMSEP (%)	Slope	Bias	RPD
GS-LS-SVM	Prediction set	2.70 ∗ 10^4^	188.59	0.95	1.47	0.98	1.24	0.98	0.07	6.96
	Verification set					0.97	1.48	0.98	−0.18	6.09

ABC-LS-SVM	Prediction set	5.00 ∗ 10^4^	49.52	0.98	1.33	0.98	1.19	0.98	−0.07	7.25
	Verification set					0.98	1.37	1.01	0.12	6.58

## Data Availability

The data used to support the findings of this study are included within the article.
